# Generation of Human Stomach Cancer iPSC-Derived Organoids Induced by *Helicobacter pylori* Infection and Their Application to Gastric Cancer Research

**DOI:** 10.3390/cells11020184

**Published:** 2022-01-06

**Authors:** Chia-Chen Ku, Kenly Wuputra, Jia-Bin Pan, Chia-Pei Li, Chung-Jung Liu, Yi-Chang Liu, Shigeo Saito, Te-Fu Chan, Chang-Shen Lin, Deng-Chyang Wu, Kazunari K. Yokoyama

**Affiliations:** 1 Graduate Institute of Medicine, Kaohsiung Medical University, Kaohsiung 80708, Taiwan; r991046@gap.kmu.edu.tw (C.-C.K.); kenlywu@hotmail.com (K.W.); r060139@gap.kmu.edu.tw (J.-B.P.); a0933836857@gmail.com (C.-P.L.); csl@kmu.edu.tw (C.-S.L.); 2 Regenerative Medicine and Cell Therapy Research Center, Kaohsiung Medical University, Kaohsiung 80708, Taiwan; pinkporkkimo@yahoo.com.tw; 3 Cell Therapy and Research Center, Kaohsiung Medical University Hospital, Kaohsiung 80756, Taiwan; ycliu@kmu.edu.tw; 4 Division of Gastroenterology, Department of Internal Medicine, Kaohsiung Medical University Hospital, Kaohsiung 80756, Taiwan; 5 Waseda Research Institute of Science and Engineering, Waseda University, Tokyo 169-0051, Japan; saict1@maple.ocn.ne.jp; 6 Saito Laboratory and Cell Technology, Yaita, Tochigi 329-1571, Japan; 7 Department of Obstetrics and Genecology, Kaohsiung Medical University Hospital, Kaohsiung 80756, Taiwan; tefu.chan@msa.hinet.net; 8 Department of Biological Sciences, National Sun Yat-sen University, Kaohsiung 80424, Taiwan

**Keywords:** stomach antral stem cells, stomach corpus stem cells, *Helicobacter pylori*, organoids, stem cell niches

## Abstract

There is considerable cellular diversity in the human stomach, which has helped to clarify cell plasticity in normal development and tumorigenesis. Thus, the stomach is an interesting model for understanding cellular plasticity and for developing prospective anticancer therapeutic agents. However, many questions remain regarding the development of cancers in vivo and in vitro in two- or three-dimensional (2D/3D) cultures, as well as the role of *Helicobacter pylori* (*H. p.*) infection. Here, we focus on the characteristics of cancer stem cells and their derived 3D organoids in culture, including the formation of stem cell niches. We define the conditions required for such organoid culture in vitro and examine the ability of such models for testing the use of anticancer agents. We also summarize the signaling cascades and the specific markers of stomach-cancer-derived organoids induced by *H. p.* infection, and their stem cell niches.

## 1. Introduction

Gastric organoids are model cell assemblages derived from gastric stem cells (GSCs) or pluripotent stem cells (PSCs). Organoids derived from long-term gastrointestinal (GI) organotypic cultures include epithelial-tissue-derived organoids [1], epithelial-tissue-derived mesenchymal organoids [2], and PSC-derived organoids [3]. Such GI organoid culture systems enable the formation of structures resembling gland crypts and microenvironments including defined niche factors, such as matrigel, Wnt, epidermal growth factor (EGF), Noggin, and R-spondin [1,2,3,4,5,6]. They stimulate the microenvironment of the GI epithelium in vitro to produce GSCs or PSCs and epithelial cells derived from them. The main cause of epithelial injury in the human stomach is *Helicobacter pylori* (*H.* *p.*) infection, which can lead to gastritis, peptic ulcers, and gastric cancers [7]. Stomach ulcers can occur when the inner wall of the stomach is irritated. The repair stage includes re-epithelialization, which mainly involves the migration and proliferation of surrounding epithelial cells, followed by regeneration when the epithelial cells form new continuous and functional glandular tissues [8]. The creation of GI organ models has allowed simulation of the stomach microenvironment in vivo [9,10]. Here, we describe the roles of GSCs, three-dimensional (3D) culture of organoids and their stem cell niches, and their potential applications in developing therapies for stomach cancers.

## 2. Potential Stem Cells and Their Niches in the Antrum and Corpus

**Figure 1:**
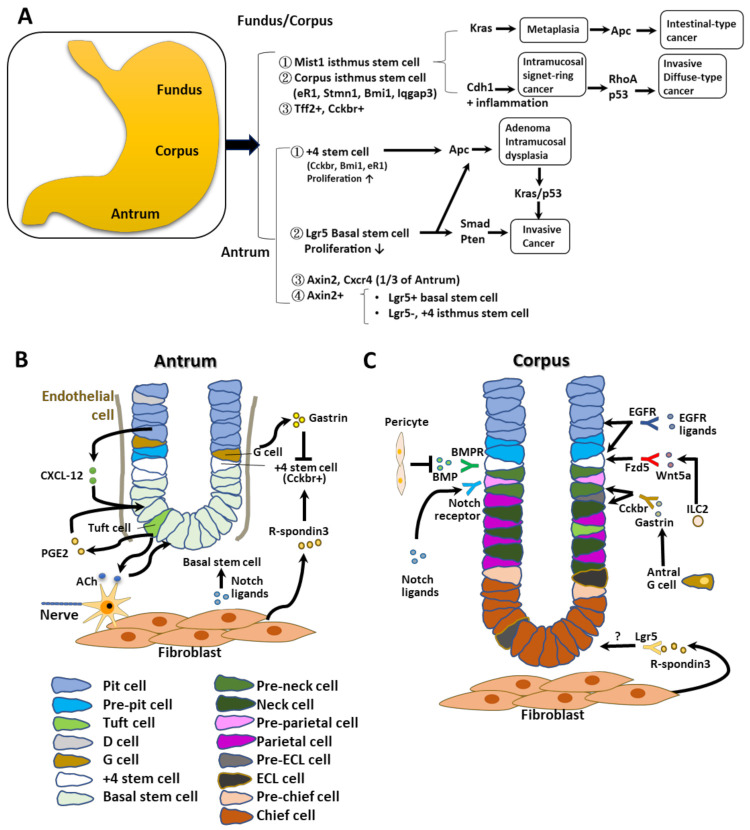
Schematic representation of gastric stem cells and stem cell niches. (**A**) A model of stem cell types reported in the stomach antrum and corpus regions. These produce metaplasia and cancers in the antrum and corpus following induction with specific oncogenic mutations. (**B**,**C**) Models of stem cell niches in the antrum (**B**) and corpus regions (**C**). Representative factors from the niches listed affect stem cells in the proliferation and development of each type. Stem cell types found in the stomach antrum and corpus regions are shown in the lower panels. Panels (**B**) and (**C**) have been modified from the original figures in Hayakawa et al. [17] with permission from Elsevier Cell Stem Cell publisher (5183941508383).

The human stomach is grouped into four subregions anatomically: the antrum, the corpus, the cardia, and the fundus (Figure 1A). It is generated from the foregut endoderm and plays an important role in the initial steps of digestion, such as the secretion of acids and enzymes. During development, interactions between mesenchymal and epithelial cells lead to the specification of cell types, pattern formation, cell differentiation, and proliferation in selected axes via respective transcription factors. In the mouse, the fundus corresponds to the foregut/cardia in humans. Human biopsy specimens and mouse recombinant strains have been used in attempts to identify the function of each stem cell type [11]. It is well known that glands from the antrum and corpus regions contain stem or progenitor cells, which generate progenies that replicate bidirectionally toward the apical and basal gland regions. This observation suggests that stem cells or progenitor cells are present primarily within the isthmic regions of glands in the antrum and corpus (Figure 1B,C) [12,13]. Both sites have immature cells with various types of granules, acting as lineage-committed progenitor cells.

In the mouse corpus, rare stem cells that are undifferentiated and granule-free are present in the gastric gland isthmus [14]. Similar types of stem cells are found in the isthmic regions of antral glands, where they can be generated bidirectionally from the stem cell zone [15,16]. Thus, both the corpus and antrum contain immature cells with different granule types thought to act as lineage-committed GSCs. Clearly, many different types of stem cells and their progenitors are present in the gastric glands.

### 2.1. Antral Stem Cells

In the LGR5+ CreERT mouse line, intestinal type Lgr5^+^ stem cells can be detected at the antral gland base (Figure 1A,B) [1,18]. (i) Such Lgr5^+^ cells have been identified in the basal regions of crypts in regions +1 to +3, below the proliferated antral gland isthmus, where they act as basal stem cells [19]. (ii) Several +4 antral stem cells have been identified in the antral gland isthmus, and are specified by markers such as Cckbr, muscle intestine and stomach expression 1 (Mist1), Bmi1, and Estrogen Receptor 1 (eR1). They have a faster growth rate than antral Lgr5^+^ stem cells [19,20,21,22,23]. (iii) In axis inhibition protein 2 (Axin2)-CreERT mice, Axin2^+^ and Cxcr4^+^ cells are present in the lower third parts of antral glands, including the isthmic and basal regions [19,21]. The Axin2^+^ cell population comprises different stem cell populations, such as Lgr5^+^ basal stem cells and Lgr5^–^ +4 isthmus stem cells. The Axin2^+^Lgr5^–^ isthmus stem cells are the major target of R-spondin [19].

Most Lgr5^+^ stem cells divide to generate two stem cells symmetrically. Thus, the numbers of Lgr5^+^ stem cells are maintained stably by neutral drift with competition. On the other hand, the +4 antral stem cells and Cholecystokinin B Receptor (Cckbr)^+^ stem cells undergo asymmetric division, with progeny expressing either numb or delta-like canonical notch ligand 1 [24].

### 2.2. Antral Stem Cell Niches

Antral GSCs interact with stem cell niches as described below (Figure 1B). (i) In part, R-spondin3 is secreted from myofibroblasts residing near the muscularis mucosae by interacting with activated Axin2^+^ +4 stem cells [19]. (ii) Antral region basal Lgr5^+^ cells, or other types, such as Aquaporin-5 (Aqp5)^+^ stem cells, are activated by Wnt signals acting through the Frizzled-7 receptor [25,26]. (iii) G cells and nearby Cckbr^+^ stem cells with gastrin receptors secrete the peptide hormone gastrin, which inhibits the growth of antral stem cells and maintains their quiescence. Gastrin deficiency causes the Cckbr^+^ stem cells to proliferate more extensively and to divide symmetrically [24]. Activation of Notch in the antral glands results in the loss of G cells and increases the gastrin-dependent proliferation of Cckbr^+^ stem cells. Deletion of the mouse gene encoding gastrin enhanced tumor development in the antrum, and antral G cells serve as important niches to repress stem cell expansion for gastric cancer progression [27]. Other receptors are also required for stromal niche signals, as follows. (iv) Antral Lgr5^+^ cells express muscarinic acetylcholine receptors controlled by cholinergic neurons, and tuft cells express choline acetyltransferase [28]. (v) Tuft cells also produce prostaglandins [29,30], secreted from fibroblasts to increase the stemness of the intestinal stem cells and GSCs [31]. Basal/isthmic antral stem cells in gland crypts have the Cxcr4 receptor, whereas Cxcl12 is generated from vascular endothelial cells adjacent to GSCs [21]. Proliferation and propagation of stem cell niches often precede stem cell expansion during tumorigenesis, which promotes the symmetric division of stem cells [24].

### 2.3. Corpus Stem Cells

Corpus stem cells have been characterized using Mist1Cre ERT2 mice as follows. (i) Mist1 (bhlha15) was regarded as a marker of gastric chief cells, which are rare corpus solitary cells. Mist1^+^ isthmus stem cells show slow cycling and division [32]. They can produce daughter cells from the isthmus region into both sides to form the entire corpus gland. Moreover, they act as truly quiescent stem cells from the corpus and contribute to the slow conversion of corpus glands. However, Mist1^+^ cells were also identified as other types, such as zymogenic or gastric chief cells. Therefore, further studies are necessary to define Mist1^+^ isthmus cells. (ii) Isthmus stem cells expressing eR1, Stathmin1 (Stmn1), Bmi1, and Ras GTPase-activating-like protein (Iqgap) have also been identified [20,22,23,33,34,35]. The isthmic Stmn1/Iqgap3-positive cells overlapped with proliferating Ki67^+^ cells. These isthmic cells produced all lineages in the corpus during development, including basal chief cells, mucous neck cells, parietal cells, and pit cells [34]. Stmn1^+^ cells were also shown to be heterogenous populations and Bmi1^+^ cells were quiescent, but, after epithelial injury, they entered a more active cycling stage. Similarly, isthmus cells, either Mist1, eR1, or Stmn1, entered a more rapidly dividing lineage to have acute damage or *Kras* gene mutations, which contributed to monoclonal cell formation [22,33]. (iii) Trefoil factor 2 (Tff2)^+^ and Cckbr^+^ cells are short-lived, lineage-committed progenitors. Cckbr expression was detected in both cell types, such as parietal cells, which secrete acids, and from enterochromaffin-like (ECL) cells that release histamines, which can mediate acid secretary responses to react with gastrin. However, such cells were also detected in the corpus isthmus, and, during hypergastrinemia, the proliferation potency of Cckbr^+^ isthmus cells increased [36]. In the normal state, Cckbr^+^ isthmus cells are the origin of expanded ECL cells that accumulate during chronic hypergastrinemia [36]. Thus, the Cckbr^+^ cells in the corpus are functionally different from those in the antrum.

### 2.4. Corpus Stem Cell Niches

Wnt, R-spondin, Noggin, and EGF are required for the long-term culture of 3D gastric corpus organoids [10,37], suggesting that these components might be present in this region, in the stem cell niche (Figure 1C). Activation of Notch promoted isthmic cell proliferation and cancer progression. Presence of the EGF receptor and its ligands and *Ras*/*Raf* mutations led to robust isthmus cell proliferation. The Noggin/BMP pathway might act to stimulate corpus stem cells. Wnt/R-spondin target genes such as *Lgr5* and *Axin2* are dominant at the base of the gland. Thus, noncanonical Wnt5-positive type 2 innate lymphoid cells (ILC2s) were found to accumulate around the stem cell area and activated isthmus stem cells [38]. Frizzled-5, a putative Wnt5a receptor, is present in gastric isthmus stem/progenitor cells. Thus, the Wnt/R-spondin axis plays a critical role in niche function in corpus stem cells.

### 2.5. Gastric Chief Cells Are Potential Reserve Stem Cells

Gastric chief cells function as the main reservoir of corpus stem cells following injury [33]. They express Lgr5, Mist1, and Troy, and, most specifically, Gpr30. However, there are several issues in interpreting the transition of chief cells into stem cells, and thus further investigation is needed. Two different progenitor zones resembling the isthmus and basal layers have been described in detail in the proximal fundus stomach [33].

## 3. Cellular Origins of Antral Gastric Cancers

**Figure 2:**
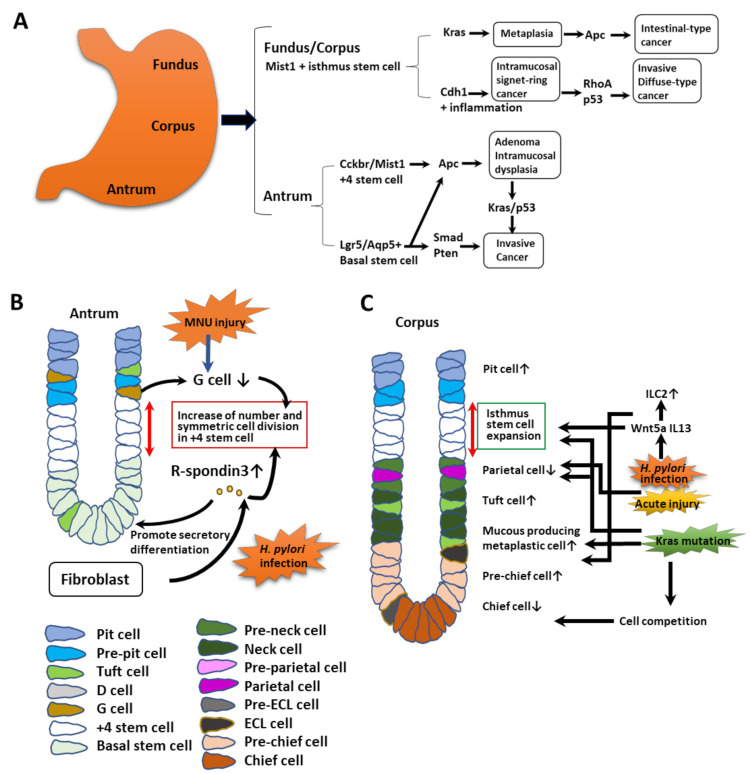
Generation of metaplasia and tumors from stem cells. (**A**) Stem cells in both the antrum and corpus generate metaplasia and cancers following oncogenic gene mutations. The combination of multiple alterations of genes results in more invasive tumorigenic cells in each region. (**B**) Alterations in antral stem cells and niches at early phase of tumorigenesis. Chronic injury following N-Nitroso-N-methylurea (NMU) treatment or *H. p.* infection changes the stem cell niches, with an increase in R-spondin secretion from fibroblasts, and acetylcholine from neurons and tuft cells in addition to the decreases in G cells and gastrin. The changes lead to the expansion and symmetric division of +4-type stem cells, differentiating to the Lgr5^+^ cell lineage. (**C**) Cellular alterations in stomach corpus glands during initial metaplasia. Following acute and chronic injuries, the isthmus progenitor cells, tuft cells, and mucus-producing metaplastic cells expand and both parietal and chief cells are reduced in number. Wnt5a and IL-13 from ILC2 cells affect these changes. *kRAS* mutations in the isthmus stem cells result in similar metaplastic changes. Such mutations in mature chief cells can result in cell-competition-dependent loss of cells. This figure was modified from the original figures published by Hayakawa et al. [17] with permission from Elsevier Cell Stem Cell publisher (5183941508383).

In the mouse antrum, both Lgr5^+^ basal stem cells and +4 stem cells can generate oncogenic mutations to cause cancer (Figure 2A,B). An adenomatous polyposis coli (*Apc)* mutation in antral stem cells resulted in adenomas or intramucosal dysplasia [1,21,39]. Combinations of multiple mutations of oncogenic driver genes including *Apc*, *p53*, *Kras*, *Pten*, or *Smad* were found to generate invasive tumors originating from antral stem cells [24,26,40]. Thus, driver mutations in these stem cells were required to initiate gastric carcinogenesis.

*H. p.* infection alters the antrum stem cell niches rapidly, with the induction of R-spondin3 production from fibroblasts, which was found to stimulate the expanding symmetric cell division of Axin2^+^ +4 stem cells in the antrum [19]. The Lgr5^+^ stem cells also proliferated later in response to R-spondin [41,42], which also promoted secretory differentiation [43]. Thus, both antral stem cells and their stem cell niches are modified and clonally expanded following exposure to carcinogenic ligands that can generate gastric cancers. In tuft cells, acetylcholine-dependent nerve signaling was increased during tumorigenesis and cholinergic innervation and generated the clonal propagation of stem-cell-derived clones. In addition, N-methyl-N-nitrourea (NMU)-induced injury activated the Notch cascade in antral regions to reduce the number of gastrin-producing G cells and increase the number of Cckbr^+^ stem cells (Figure 2B) [24].

## 4. Oncogenic Mutations in Corpus Isthmic Stem Cells

Corpus stem cells are known to be more resistant to oncogenic transformation than antral stem cells [44]. Single mutations in the corpus stem cells are insufficient to generate dysplasia or tumors. For example, following deletion of the *Cdh1* gene, Mist1 stem cells underwent programmed cell death in the form of anoikis, and *Cdh1*-deficient Mist1-derived clones reduced gradually in number. However, following the chronic inflammation produced by *H. p.* infection, *Cdh1*-deficient Mist1-derived clones were able to survive and expand, and then eventually caused cancers, which might resemble human signet ring cell gastric cancers [44]. In part, this might have been in response to an increased level of the Cxcl12 protein and expansion of ILC2 cells through the action of Wnt5a (Figure 2A,C).

Additional gene mutations of *p53* or *Rhoa* in *Cdh1*-deficient Mist1^+^ stem cells accelerated the formation of diffuse-type cancers [44] (Figure 2A,C). Although the *Apc* mutation in corpus stem cells was not able to induce tumorigenesis at this site by itself, simultaneous mutations in the *Kras* and *Apc* genes resulted in producing gastric cancers efficiently. Thus, *H. p.* infection and *Kras* mutation in the stomach result in gastric atrophy, leading to the loss of both parietal and chief cells and subsequent metaplasia. Such pathological abnormalities are essential to form cancers derived from isthmic stem cells in the corpus. It is known that the loss of parietal cells induces an increase in isthmus-derived clones and gastric atrophy, which precedes tumorigenesis in humans [33]. Therefore, it is possible that stem cells from the glandular isthmus act as the main source of gastric cancers after interactions with other stem cells and their niches.

## 5. Gastric Organoid Culture Systems and Microenvironments

### 5.1. Effects of Extracellular Matrix (ECM) and Wnt/R-Spondin

Matrigel is a soluble form of basement membrane [45], rich in extracellular proteins including laminin, collagen V, heparan sulfates, entactin/nidogen, and some growth factors, including transforming growth factor-beta (TGF-β) and fibroblast growth factor (FGF) [46]. It can mimic the natural basement membrane. Tight junctions between epithelial cells over the basement membrane are critical for their survival via integrin signaling. In the case of integrin-mediated basement membrane alterations, the loss of attachment can induce cell apoptosis [47]. Detachment-induced cell death was also observed when contact with the epithelial blood supply was disrupted, and this was named anoikis. When culturing isolated single intestinal stem cells, the inhibition of Rho kinase (ROCK) resulted in the prevention of anoikis [47].

Intestinal organoids used to be cultured in type I collagen gel, but the plating efficiency was lower than among various organoids maintained in matrigel, indicating that the intestinal epithelium is beneficial to the ECM [46]. The characteristics of epithelial and epithelial/mesenchymal organoids generated by stomach tissues or PSCs/iPSCs derived from the stomach, small intestine, and colon are shown in Table 1. The components of culture media for the antrum and fundic organoids as well as human and mouse organoids are listed in Table 2 [48,49]. Gastric organoids can be generated from normal and cancerous tissues. Patient-derived gastric organoids are generally prepared from surgical tumor specimens or endoscopic biopsies [50]. Small tissue fragments (approximately 2–5 mm^3^) were suspended in matrigel-containing culture medium supplemented with essential components including EGF, Noggin, R-spondin, Wnt, FGF, TGF, and gastrin. These supplements make 3D organoids and culture niches very complex and different from conventional two-dimensional (2D) culture media. This is a major problem, leading to contamination with epithelial and stromal cells, and cancer cells when the tissue fragments are used as the source of 3D organoids [37,51]. By contrast, iPSC- or ESC-derived organoids are genetically well defined to generate all cell types and are easily manipulated by gene editing and similar techniques. Thus, PSC-derived human gastric organoids have been beneficial for studying the complex GI epithelium with its glandular architecture and surrounding niches [3]. Therefore, PSC-derived organoids serve as great models for studying the mechanisms underlying human digestive diseases to facilitate drug discovery. However, there is still a problem that PSC-derived organoids show limited cell maturation, with features resembling fetal rather than adult tissues [3,52].

**Figure 3:**
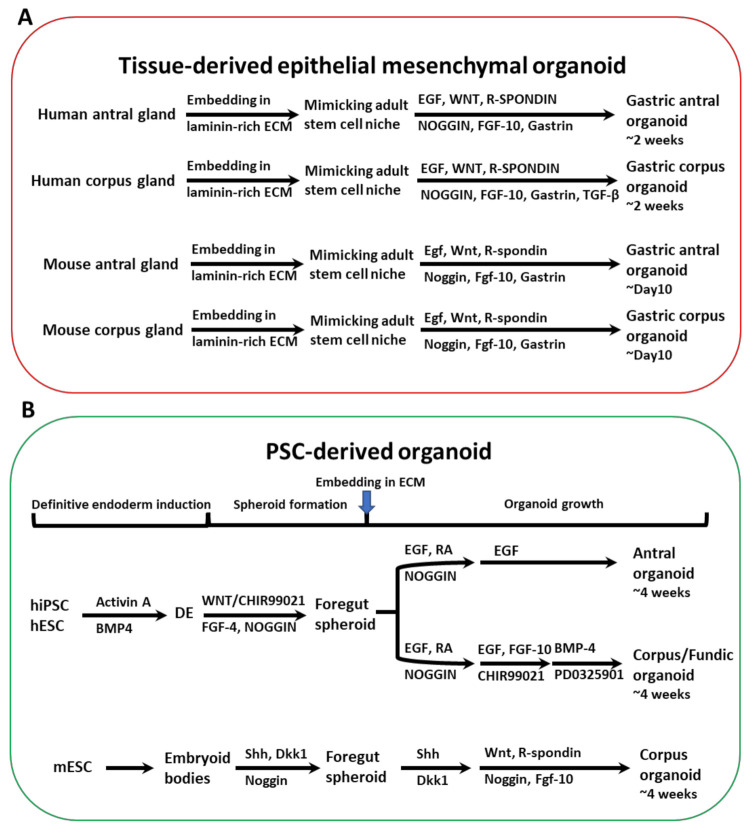
Generation of stomach organoids from tissue and pluripotent stem cells. (**A**) Mouse or human stomach antrum and corpus organoids were generated from the glands, embedded in extracellular matrix (ECM), and cultured with medium supplemented with EGF, WNT, R-spondin, Noggin, FGF10, and gastrin. Suppression of TGF-β signaling increased the longevity of human stomach corpus organoids. (**B**) Differentiation of pluripotent stem cell (PSC)-derived human antrum and corpus organoids. PSCs were isolated from blastocysts (embryonic stem cells; ESCs) or reprogrammed to generate iPSCs. Cells were committed to differentiate into endoderm by treatment with Activin A and BMP4. Posterior foregut formation was achieved by culture with FGF4 and Wnt or CHIR99021. Noggin was inoculated in the case of foregut differentiation. ECM-embedded cells produced the 3D foregut spheroids. Differentiation into antral types was done using retinoic acid (RA) and EGF treatment. To produce foregut cell types, the organoids were exposed to CHIR99021, EGF, and FGF10. Mouse PSC-derived corpus organoids were generated by culturing the embryoid bodies and treatment with Sonic hedgehog (Shh), the WNT antagonist Dickkopf 1 (Dkk 1), as well as Noggin. ECM-embedding spheroids with exposure with FGF10, Noggin, WNT, and R-spondin produced corpus gland formation in mice [47,48].

**Figure 4:**
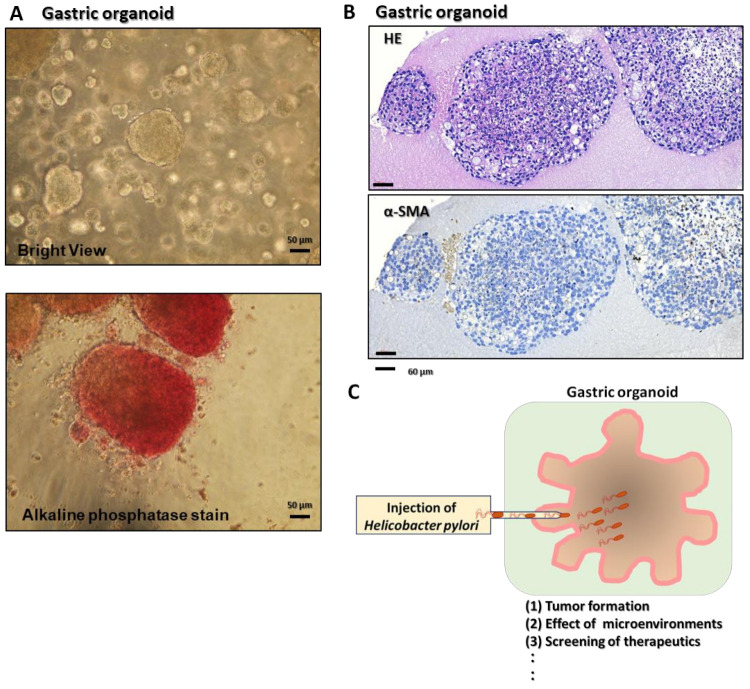
Alkaline phosphatase staining and immunohistochemistry of gastric organoids in matrigel-containing 3D cultures. (**A**) Bright-field view of an adenocarcinoma from human stomach-derived organoid lines (upper panel). HCM-BROD-0208-C16 cancer model primary adenocarcinoma cells of the stomach were cultured using a recommended American Type Culture collection protocol [6]. The stomach organoids were generated by seeding in 96-well plates and grown for 2 days (*n*  =  6). The organoids were fixed with 4% paraformaldehyde, and incubated with alkaline phosphatase detection reagents (SCR004, Millipore, Merck KGaA, Darmstadt, Germany). Positive alkaline phosphatase staining, a characteristic stem cell feature, is shown in red (lower panel). Scale bars = 50 μm. (**B**) Gastric organoids at the maturation stage were generated in regular matrigel-containing 3D culture, and gastric organoids were verified by hematoxylin and eosin staining (upper panel). Alpha-smooth muscle actin (lower panel) was used as a marker of pericryptal myofibroblasts in gastric cancers. Scale bars = 60 μm. (**C**) Schematic model of proposed experiments using the gastric organoids injected by *H. p*. are presented. (1), (2), (3),--; these functions will be examined.

R-spondin was found to be expressed and secreted in intestinal subepithelial fibroblasts [65]. After R-spondin was bound to the Lgr5 receptor, it inhibited cell surface transmembrane E3 ubiquitin ligase Zinc and Ring Finger 3 (ZNRF3) and its functional homolog Ring finger protein 43 (RNF43) RNF43/ZNRF3-mediated breakdown of Wnt receptors and continued the upregulation of *Wnt* expression. It is well known that such organoids need Wnt and R-spondin for culture and reproduction [66,67,68]. However, mouse small intestine organoids contain Paneth cells that produce Wnt and can grow without adding exogenous Wnt ligands. Lgr5^+^ cells are found together with Paneth cells in organoids, and the differentiation of intestinal cells eventually occurs in cyst areas lacking Paneth cells. However, the culture of mouse colon organoids and human small intestine/colon organoids requires the addition of exogenous Wnt ligands. This might be because of insufficient endogenous Wnt production [4,69]. Intestinal subepithelial fibroblasts produced Wnt ligand and R-spondin, and coculture with fibroblasts allowed organoids to grow without exogenous Wnt ligands or R-spondin [65,70]. However, APC deficiency or constitutively active mutations in the gene for β-catenin conferred Wnt/R-spondin-independent growth in intestinal organoids [71]. Glycogen synthase kinase 3 beta (GSK-3β) inhibitors such as CHIR99021 replaced the Wnt/R-spondin ligands in mouse intestinal organoid cultures [72]. However, these compounds are generally toxic to human intestinal organoids [73]. Therefore, it is necessary to use appropriate doses of these reagents. Togasaki et al. reported that Wnt 3a- and R-spndin1-depleted cultured organoids formed loosely connected clusters of swollen cells and showed features of signet-ring cell carcinomas [74]. A ROCK inhibitor, a GSK inhibitor, and Wnt 3a were critical for the growth of gastric cancer organoids, although the requirement for R-spondin was the same between normal and cancerous cells [75]. Stem-cell-derived organoids were also developed using a standard protocol as for embryonic gastric tissue development [6]. The PSC-derived organoids were differentiated into a definitive endoderm by exposure to activin A; then, the addition of FGF4 generated both the anterior and foregut endoderm. The endoderm cells were then exposed to inhibitors of GSK3β, such as CHIR-99-21, and inhibitors of BMP, such as Noggin. The foregut spheroids were committed to the posterior foregut by treatment with retinoic acid (RA), whereas organoids composed of endocrine cells—such as G cells—could be generated by treatment with EGF, Noggin, and RA for 4 weeks. By contrast, fundic stomach organoids were formed by adding a GSK-3β inhibitor, and a mitogen-activated protein kinase kinase inhibitor, BMP4, was required for the induction of differentiated parietal cells. These organoids were mixed and cultured with stem cell types including endothelial, immune, mesenchymal, and neuronal cells to identify the critical components for generating human gastric organoids (Figure 3). To examine the effect of the microenvironment of gastric cancer development to give the critical signaling to gastric cancer CSCs, this organoid model is very useful. As shown in Figure 4, we prepared human gastric cancer organoids as the starting materials and examined the tumorigenicity, the effect of stem cell niches, signaling induced by *H. p.* infection, and the development of the new therapeutics. These were stained for alkaline phosphatase as the stem cell marker, and alpha-smooth muscle actin, a marker of procryptic myofibroblasts (Figure 4). In addition, these technologies are now being used for the analysis of model diseases [3,10,76]. This organoid technology is also being applied to drug screening, transplantation, and CRISPR-mediated genomic editing for genetic diseases including cancers [70].

### 5.2. Niche Microenvironments

The niche microenvironments comprise stem cells, stromal cells, and immune cells. Interactions between stem cells and these niches initiate the proliferation and differentiation of the stem cells. However, the microenvironments of gastric stem cells have not been clarified well because the gastric glands are complex, and their cellular plasticity has not been studied in detail.

The epithelial cell lineage of the GI system is generated from common embryonic endoderm stem cells [77] and exhibits constant renewal to maintain homeostasis in the adult. The epithelial transition from undifferentiated cells involves LGR5 signaling to form the mature intestinal epithelium, indicating that stem cell identity is an inducible form of plasticity [78]. The development of the intestinal niche is governed by EGF, WNT, and R-spondin signals in the glandular crypts to enhance cell replication, while BMP signals at the apical tips of villi help with cell differentiation [79,80]. Notch signaling is also critical for this [81]. Proliferation of Lgr5+ antral stem cells was achieved by inhibiting differentiation via Notch 1 and Notch 2 receptors [62,82]. The mesenchymal compartment surrounding the glands is not well clarified. Cxcl12^+^ endothelial cells and Cxcr4^+^ innate lymphoid cells were shown to contribute to the corpus stem cell niche [44]. In addition, R-spondin-3 secreted from myofibroblasts plays a critical role for antral stem cell niches and predominantly activated Axin2^+^/Lgr5^–^ stem cells. Gastrin from G cells residing in the antral isthmus region, and CCK2R as the receptor for gastrin, are also important. CCK2R^+^ stem cells have been found in the same zone. Progastrin can stimulate the proliferation of CCK2R^+^-expressing stem cells, but gastrin does not have such activity [83]. Acetylcholine from tuft cells in the gastric epithelium regulated the proliferation and regeneration of the epithelial as well as clonal expansion of Lgr5^+^ stem cells [42]. These growth factors and cellular components are critical for maintaining gastric stem cell niches; however, the cells providing these niche factors and their interactions remain elusive.

### 5.3. H. p. Infection

In mouse models of *H. p.* infection, atrophy and metaplasia are detected in the progression of gastric cancer. In humans, *H. p.* infection induces classic intestinal metaplasia (IM), pseudopyloric metaplasia, leading to spasmolytic polypeptide-expressing metaplasia (SPEM). IM barely developed even after long-term *H. p.* infections in mice. Therefore, the origin of IM in the human stomach remains unknown. However, assuming that IM expands as a monoclonal cell lineage, long-lived stem cells are possibly the source. SPEM together with chronic atrophic gastritis and IM are pathological signs of long-term *H. p.* infection; so, they might reflect mutations or epigenic changes in GSCs. During SPEM regeneration, the cell types in the corpus glands change significantly, with the disappearance of chief and parietal cells, as well as the expanded growth of progenitor cells of isthmus and tuft cells. Type 2 innate lymphoid (ILC2) cells, important components of the corpus stem cell niches, are present in some pathological alterations induced by the secretion of Wnt5a and interleukin-13 (IL-13) [84,85]. These metaplastic regions are linked with a higher risk of upper GI cancers. Indeed, some reports instead have shown the suppression of tumor formation in SPEM lesions enriched with TFF2 and MUC6 molecules [86,87]. Thus, it remains to be confirmed whether SPEM is a real precursor of IM or cancer.

### 5.4. Hepatoma-Derived Growth Factor (HDGF) and Tumor Necrosis Factor (TNF)-α Are Required for H. p. Infection

HDGF, isolated from the conditioned medium of cultures of human hepatocellular carcinoma cell line Huh-7, is an acidic heparin-binding growth factor [88]. The human HGDF protein comprises 240 amino acids with a bipartite nuclear localization motif [89,90] and a PWWP domain [91,92]. Thus, HDGF might have dual functions as a cytokine and a transcription factor [90,93]. *H. p.* colonizes the gastric epithelium and increases the risk of gastric cancer [94]. In H^+^, K^+–^Noggin transgenic mice, *H. p.* infection can further increase the levels of inflammatory cytokines such as TNF-α, interferon gamma, MIP-2, and IL-1β [95]. Conversely, treatment with BMP2, BMP4, and BMP7 reduced TNF-α-induced IL-8 expression [95]. *H. p.* infection also promoted the expression of HDGF in human gastric cancer cells [96,97]. Stem cells treated with HDGF exhibited a carcinoma myofibroblast phenotype, which promoted cell survival and the invasion of human gastric cancer cells [97]. *H. p.* infection triggered an inflammatory TNF-α/HDGF/cyclooxygenase-2 (COX-2) pathway in the stomach and induced gastric carcinogenesis, and HDGF overexpression caused gastric inflammation and carcinogenesis [96]. HDGF levels in patients with *H. p.* infections or IM in precancerous lesions were increased significantly, which promoted the infiltration of neutrophils and transmitted *H. p.*-induced inflammatory signals. In a mouse model, knockout of the gene for HDGF significantly inhibited *H. p.*-induced neutrophil infiltration and inflammation by TNF-α/COX-2 signaling, thereby alleviating gastric damage [96]. A time-course study indicated that TNF-α acts as a priming factor to increase HDGF release from hepatoma cells during *H. p.* infection, and HDGF is also known as an intermediator that amplifies and maintains downstream COX-2/TNF-α signaling through the activation of nuclear factor kappa B (NF-κB) [96,97]. To understand this issue in detail, we have investigated the close relationship between *H. p.* infection and HDGF/TNF-α signaling using organoids from normal human gastric tissues and from gastric cancers [96].

## 6. Conclusions

We have summarized information on gastric cancer stem cells and their organoids to identify distinct markers for the induction of human gastric cancers that interact with stem cell niches. It has been shown that *H. p.* infection activates NF-κB-dependent inflammation in gastric epithelial cells and stimulates the formation of the chemokine IL-8 [98] and its virulence factor CagA, forming a complex with the MET receptor and activating the proliferation of epithelial cells [99,100]. These functions were reproduced in antral organoid cultures derived from human ESCs [3] or primary human stomach corpus region biopsies [10]. Both Wnt ligands and R-spondin have also been reported as niche factors not only for the normal GI epithelium but also for gastric cancers [74]. Sigal et al. also reported that *H. p.* infection triggered the expression of R-spondin3 in gastric fibroblasts [19]. Thus, host–microbial interactions during tumor development might contribute to histopathology subtype specifications. Mutations of *TP53* could induce a reversal to a poorly cohesive carcinoma not otherwise specified, and these cancers re-expanded after the addition of WNT signaling [101]. The genetic mutations and the alterations of the tumor environment can be critical for gastric cancer progression. These fields should be investigated further to clarify the identification of new biomarkers for triggering and developing stomach cancers.

Numerous questions remain unanswered, as follows. First, single-cell analysis has revealed extensive heterogeneity in the presumed gastric stem/progenitor cell populations. Therefore, the sorting and separation of individual stem cells is necessary to study their functional characteristics in detail. The second question is how cancerous stem cells can develop to form metaplasia. It seems that the involvement of genetic and epigenetic changes might be crucial. Single-cell analysis and single-cell ATAC sequencing are required to understand this issue. The interactions of cancer stem cells with their niches are also critical for tumorigenesis. Thus, the generation of human GI organoids and humanized animal models would facilitate an understanding of the commitment to form gastric cancers. The third question involves cellular plasticity. Dedifferentiation and interconversion from progenitors and stem cells involve tissue regeneration. To understand this question, understanding the interactions between stem cell-derived organoids and stem cell niches is critical. In these circumstances, combined organoid methods might be interesting to clarify dedifferentiation and the interconversion processes for further study. Combining organoids derived from different tissues and environments such as gastroenteropancreatic neuroendocrine neoplasms can contribute to 2D and 3D organoids with their environments, which could provide useful links between cancer stem cells and research on the microenvironment of the development of gastric cancers [102]. Using these technologies, we have developed therapeutic agents that could act during cancer development using stem-cell-derived organoids and niches. Thus, we have focused on GSCs in detail to understand their interactions with the niches induced by internal and external cancer drivers such as *H. p.* and other mycobacteria and chemicals. In fact, the stomach organoids, combined with intestine organoids, colon organoids, and mesenchymal organoids, will be beneficial for study of the gastrointestinal microbiota together with *H. p*. interaction and luminal physiology in future works.

## Figures and Tables

**Table 1:** Long-term culture of gastrointestinal organoids. Organoids from gastric epithelial cells or epithelial and mesenchymal cells are compared. The characteristics of gastrointestinal organoids are summarized.

	Epithelial Only Organoids	Epithelium and Mesenchymal Organoids	
	Tissue-Derived Epithelial	Tissue-Derived Epithelial Mesenchymal Organoids	PSC-Derived Organoids
Characteristics	One Cell Layer	Grown in Air–Liquid Interface	Human Fetal Gastrointestinal Tissue Generated From iPSCs or ESCs
**Stomach**	Barker 2010 [1] DeWard 2014 [53]	Katano 2013 [2] Miyoshi 2013 [54] Li 2014 [55]	McCracken 2014 [3] Hannan 2013 [56]
**Small intestine**	Sato 2009 [4] Stange 2013 [37]	Ootani 2009 [57] Li 2014 [55]	Spence 2011 [52] Forster 2014 [58] Hannan 2013 [56] McCracken 2011 [59]
**Colon**	Sato 2011 [60] Yui 2012 [46]	Li 2014 [55]	Crespo 2017 [61]

**Table 2:** Characterization of mouse and human gastric organoids. Each culture component in the organoid medium is compared and listed. Tissue-derived and induced pluripotent stem cell (iPSC)- and embryonic stem cell (ESC)-derived organoids are compared in terms of the precise components of their culture media. Key: APT, inhibitor of the γ-secretase complex; DKK1, Dickkopf1; FBS, fetal bovine serum; HEPES, 4-(2-hydroxyethyl)-1-piperazineethanesulfonic acid; NEAA, nonessential amino acids; SHH, Sonic hedgehog.

	Tissue-Derived				iPSC-/ESC-Derived			
	Human		Mouse		Human		Mouse	
	Antral organoid	Fundus/ Corpus Organoid	Antral Organoid	Corpus Organoid	Antral Organoid	Fundus/Corpus Organoid	Antral Organoid	Corpus Organoid
Reference	Gifford 2017 [62]	Bartfeld 2015 [10]	Barker 2010 [1]	Stange 2013 [37]	McCracken 2014 [3] Broda 2019 [6]	McCracken 2017 [63] Broda 2019 [6]		Noguchi, 2015 [64]
Medium composition								
Basel medium	Advanced DMEM/F12	Advanced DMEM/F12	Advanced DMEM/F12	Advanced DMEM/F12	Advanced DMEM/F12	Advanced DMEM/F12		DMEM/F12
L-glutamine	2 mM				2 mM	2 mM		
GlutaMAX		1X						
HEPES		10 mmol/L			10 μM	10 μM		
B27		1X	1X	1X	1X	1X		1X
N2			1X	1X	1X	1X		1X
FBS	10%							
KSR								1%
NEAA								0.1mM
N-acetylcysteine		1 mmol/L	Acetylcysteine (Invitrogen)	Acetylcysteine (Invitrogen)				
WNT5A						50 ng/mL		
WNT3A	50% L-WRN *	50%	50%	50%				100 ng/mL
R-spondin		10%	1 µg/mL	1 µg/mL				250 ng/mL
Noggin		10%	100 ng/mL	100 ng/mL	200 ng/mL			100 ng/mL
BMP4						50 ng/mL		
EGF		50 ng/mL	50 ng/mL	50 ng/mL	100 ng/mL	100 ng/mL		50 ng/mL
FGF10		200 ng/mL	100 ng/mL	100 ng/mL		50 ng/mL		100 ng/mL
IGF		100 ng/mL						
Prostaglandin E (PGE)2		500 nmol/L						
P38i (SB202190)		10 µmol/L						
Retinoic acid					2 μM			
Nicotinamide		10 mmol/L						
TGF-betai (A-83-01)		2 µmol/L						
Gastrin		1 nmol/L	10 nM	10 nM				
Y-27632	10 μM	10 µmol/L	10 μM	10 μM	10 μM	10 μM		
GSK-3betai CHIR-99021		3 µmol/L				2 μM		
SB431542	10 μM					10 μM		
PD0325901						2 μM		
DAPT						1 μM		
Dexamethasone						50 nM		
Matrigel	Corning Matrigel	BD Matrigel	BD Matrigel	BD Matrigel	BD Matrigel	BD Matrigel		BD Matrigel
Lgr5+	yes	yes	yes	yes	yes	-		yes
Troy+	-	yes	-	yes	-	-		yes
				Definitive endoderm				
Basal medium					RPMI 1640	RPMI 1640		DMEM high-glucose
Defined FBS					0%, 0.2%, 2%	0%, 0.2%, 2%		
KSR								15%
NEAA								0.1mM
Activin A					100 ng/mL	100 ng/mL		
BMP4					50 ng/mL	50 ng/mL		
WNT3A					500 ng/mL			
FGF4					500 ng/mL	500 ng/mL		
Noggin					200 ng/mL	200 ng/mL		
DKK1								500 ng/mL
SHH								500 ng/mL
Retinoic acid					2 μM	2 μM		
CHIR-99021					2 μM	2 μM		

* L-WRN: L cell line expressing Wnt3a, R-spondin, Noggin (L-WRN).

## Data Availability

Please contact the corresponding author for such requests.
